# Underestimated Risks of Infantile Infectious Disease from the Caregiver’s Typical Handling Practices of Infant Formula

**DOI:** 10.1038/s41598-019-46181-0

**Published:** 2019-07-05

**Authors:** Tae Jin Cho, Ji Yeon Hwang, Hye Won Kim, Yong Ki Kim, Jeong Il Kwon, Young Jun Kim, Kwang Won Lee, Sun Ae Kim, Min Suk Rhee

**Affiliations:** 1 Department of Biotechnology, College of Life Sciences and Biotechnology, Korea University, Seoul, 02841 South Korea; 2 Human Milk R&D Team, Maeil Dairies, Co., Ltd, Pyeongtaek, 17714 South Korea; 3 Department of Integrated Biomedical and Life Science, College of Health Science, Korea University, Seoul, 02841 South Korea; 4 Department of Food and Biotechnology, College of Science and Technology, Korea University, Sejong, 30019 South Korea; 5 Department of Food Science and Engineering, Ewha Womans University, Seoul, 03760 South Korea

**Keywords:** Applied microbiology, Pathogens, Policy and public health in microbiology

## Abstract

The impact on infant caregiver as a reservoir of pathogens has not been exploited with perspective to powdered infant formula (PIF). Here we reveal novel route of pathogen transfer through hand-spoon-PIF unexpectedly occurred by even typical practices of caregivers, handling of PIF and storage of feeding-spoon in PIF container. Hand-spoon-PIF contamination route was simulated to analyze the transfer and subsequent survival of pathogens. Major pathogens associated with infantile fatal diseases (*Cronobacter sakazakii*, *Salmonella enterica*, *Staphylococcus aureus*) were readily transmitted to PIF from skin (3−6 log CFU/hand) via spoons following long-term survival of transferred pathogens (3 weeks; use-by date of PIF) as the excessive level of infectious dose, highlighting direct onset of diseases. Low bacterial load on skin (*ca*. 1 log CFU/hand) could prevent cross-contamination of PIF, however, at least 72 h survival of transferred pathogen on spoons demonstrated the probability on re-contamination of PIF. To our knowledge, this is the first study to investigate the cross-contamination of utensils in contact with powdered-foods. Bacterial load on hands is the key determinant of pathogen transfer and the extent of risk are species-dependent. These evidential results redefine risk of caregivers’ practices and facilitate incorporation of cross-contamination into risk-assessment as underestimated route of infection.

## Introduction

Infants have a developmentally premature immune system and gastrointestinal tract, which raises the possibility of bacterial infection when exposed to contaminated foods^1,2^. Clinical cases of foodborne illness in infants have been linked to powdered infant formula (PIF) contaminated with pathogens, especially *Cronobacter sakazakii*, *Salmonella enterica*, and *Staphylococcus aureus* for several decades^3–5^. Although bacterial contamination in PIF has been reported^6,7^ and extrinsic contamination during the handling of PIF has been believed to cause bacterial infections in infants^8,9^, the route of bacterial transmission is not yet fully understood. Previous studies have focused on the contamination of PIF during manufacturing or from raw materials (i.e. intrinsic contamination)^10^.

Practices of environmental health regarding the preparation of food have been linked to foodborne illnesses^11^. Unhygienic practices of caregivers during the preparation of PIF is speculated to be an extrinsic contamination source, but experimental verification is lacking. Food safety authorities have provided guidelines regarding the safe handling of PIF for caregivers, but failure to comply ensures extrinsic contamination remains a possibility. Guidelines recommend that caregivers wash their hands with soap before handling PIF, and sterilize feeding utensils thoroughly between uses^12,13^. However, previous surveys reported that caregivers often handle PIF poorly, fail to follow guidelines for hand sanitizing, and store spoons in the PIF container without washing, resulting in contact between PIF and spoons that have been touched directly by caregiver’s hands. According to a consumer survey (n = 1,469) on caregivers’ behavior, 55% caring for infants aged 1.5–4.5 months did not wash their hands with soap before reconstitution of PIF^14^. Hong^15^ reported that most caregivers (88%, n = 2,532) tend to store spoons in the PIF container. Observational research by Herbold and Scott^16^ reported that caregivers even put their fingers directly into PIF to retrieve spoon from the canister. Given that such inadequate practices can potentially transfer pathogens from hands or feeding utensils to PIF, assessing the probability of cross-contamination is essential to establish practical recommendations for caregivers.

Although cross-contamination through the caregiver-spoon-PIF can be a direct exposure route of pathogens from caregiver to infants, relevant studies are limited to surveys and observational researches without the analysis of actual risk^9,14,16^. Since caregivers and utensils are bacterial reservoirs^17–19^, they facilitate pathogen transfer to foods through direct/indirect contact. Indeed, foodborne illnesses have been attributed to feeding utensils such as bottles, blenders and spoons^20,21^. However, cross-contamination of powdered-foods and simulation of PIF handling practices to explore extrinsic contamination has not been reported^22^.

We hypothesized that if spoons are handled with contaminated hands, pathogens may be transferred to spoons and subsequently to adjoining PIF. Major pathogenic bacteria which should be adopted for the comprehensive evaluation of cross-contamination during PIF handling and the probability of pathogen exposure to infants were used as target pathogens in this study. *Cronobacter* and *Salmonella* are categorized as “clear evidence of causality” (category A) by the Food and Agriculture Organization (FAO) of the United Nations and the World Health Organization (WHO)^23^. Among microbes linked to outbreaks from PIF, *S. aureus* has a high probability of cross-contamination due to high prevalence on hands^4,24,25^.

**Figure 1:**
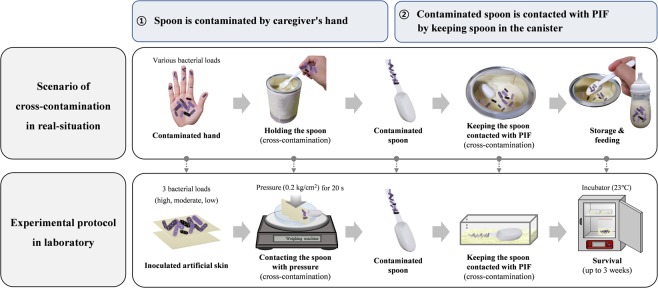
Schematic representation of cross-contamination scenarios during reconstitution and storage of PIF and experimental protocol to simulate the scenarios.

Herein, we conducted simulative analysis based on the hypotheses that if spoons are handled with hands, pathogens may be transferred to spoons and subsequently to adjoining PIF when spoons are stored in the container of products (Fig. 1). Cross-contamination of major pathogens was evaluated from the skin surface at high, moderate and low bacterial load. To examine the risk of infection and repetitive contamination, survival of transferred pathogens on spoons or PIF was also investigated. The aims of the present study were (1) to prove bacterial transfer from contaminated skin to spoons, and subsequently to PIF; (2) to analyze the effect of bacterial load on the skin surface as a determining factor of cross-contamination and subsequent survival; and ultimately (3) to construct a research base for extrinsic contamination of PIF to establish guidelines regarding proper practices for caregivers.

## Results

### Cross-contamination and survival during handling and storage of PIF following contact with a high bacterial load on skin

The population level of cross-contaminated bacteria and transfer ratio from artificial skin to spoons, and subsequently to PIF, are shown in Table 1. The initial bacterial load on artificial skin was 5.8−5.9 log CFU/hand. During simulation of the reconstitution of PIF, spoons were readily contaminated by artificial skin (transfer ratio = 24.5−33.9%), and the populations of *C. sakazakii*, *S*. *enterica*, and *S. aureus* on the spoons were 5.3, 5.2, and 5.5 log CFU/spoon, respectively. Subsequent cross-contamination to PIF from spoons was also evident, resulting in population levels of 3.7, 3.3, and 3.6 log CFU/PIF for *C. sakazakii*, *S*. *enterica*, and *S. aureus*, respectively (transfer ratio = 1.5−3.5%). Bacterial populations were not significantly different among bacterial species (*p* > 0.05) except for those on the spoons. There were no significant differences between transfer ratio from artificial skin to spoons, and from spoons to PIF (*p* > 0.05).

**Table 1:** Transfer of bacteria from artificial skin to spoon and powdered infant formula (PIF).

Bacterial load	Bacteria	Bacterial population (No. of positive samples)^x^		
		Artificial skin	Spoon	PIF
High load (6 log CFU/hand)	*Cronobacter sakazakii*	5.8 ± 0.3	5.3 ± 0.2^a,b^	3.7 ± 0.3
	*Salmonella enterica*	5.8 ± 0.1	5.2 ± 0.2^a^	3.3 ± 0.3
	*Staphylococcus aureus*	5.9 ± 0.1	5.5 ± 0.1^b^	3.6 ± 0.1
Moderate load (3 log CFU/hand)	*Cronobacter sakazakii*	3.0 ± 0.1	2.4 ± 0.2	<1.7 (20/20)
	*Salmonella enterica*	2.9 ± 0.2	2.3 ± 0.2	<1.7 (17/20)
	*Staphylococcus aureus*	2.9 ± 0.1	2.4 ± 0.2	<1.7 (19/20)
Low load (1 log CFU/hand)	*Cronobacter sakazakii*	1.0 ± 0.3	<0.7 (19/20)	<1.7 (3/20)
	*Salmonella enterica*	1.0 ± 0.2	<0.7 (18/20)	ND^y^
	*Staphylococcus aureus*	0.9 ± 0.2	<0.7 (19/20)	<1.7 (2/20)

Data in each bacterial load denoted by different alphabet are significantly different (*p* < 0.05).

^x^Results are expressed as the mean ± standard deviation (quantitative microbiological analysis; n = 6), or the positive samples/enriched samples (qualitative analysis; n = 20). Bacterial population is presented as log CFU/hand for artificial skin and log CFU/spoon for spoon, and log CFU/PIF for PIF.

^y^ND: Not detected.

**Figure 2:**
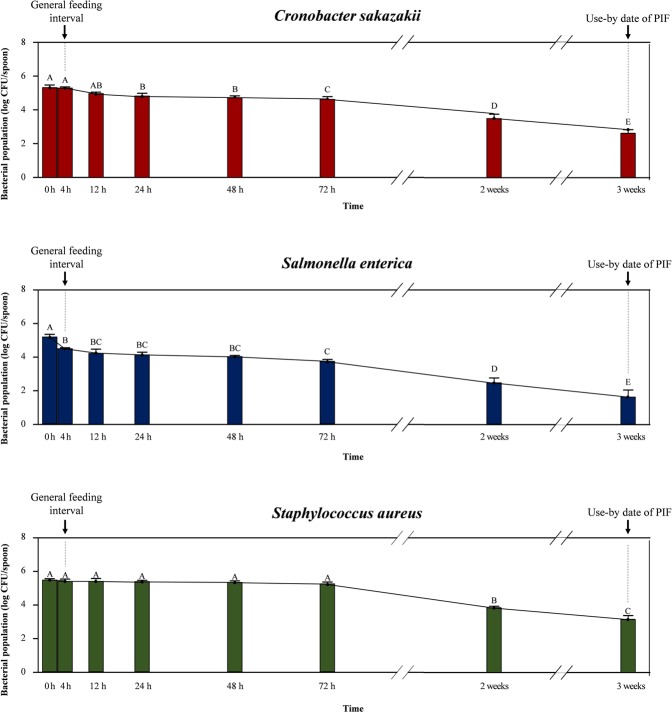
Survival of bacteria (log CFU/spoon) on the surface of spoon contaminated from artificial skin with high bacterial load (6 log CFU/hand) during storage in touched with PIF. Data showed mean ± standard deviation (quantitative analysis; n = 6). Different capital alphabets (**A**–**E**) denoted on each data indicate significant differences of population levels between experimental time points within the same bacterial species (*p* < 0.05). The arrow bars (↓) indicate the general feeding interval (4 h) and use-by date of PIF suggested manufacturer (3 weeks).

**Figure 3:**
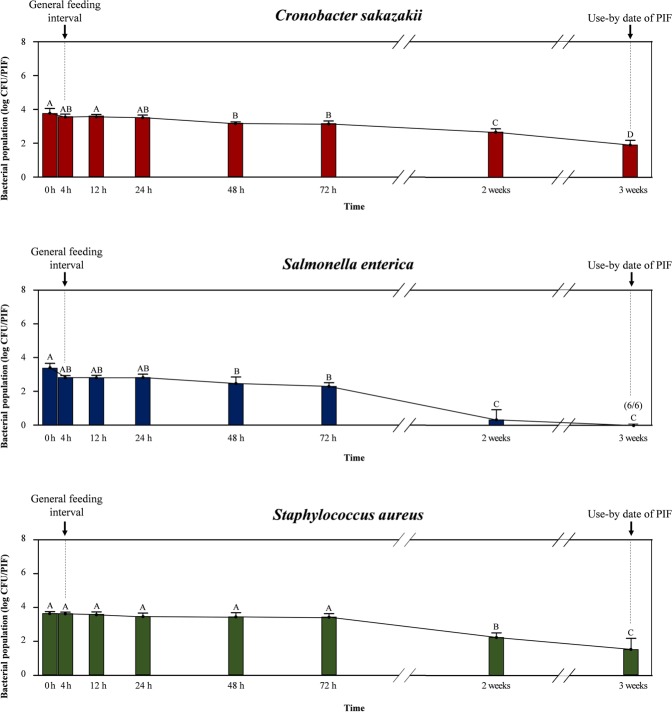
Survival of bacteria (log CFU/PIF) in powdered infant formula during storage with spoon contaminated from artificial skin with high bacterial load (6 log CFU/hand) during storage. Data showed mean ± standard deviation (quantitative analysis; n = 6). Different capital alphabets (**A**–**D**) denoted on each data indicate significant differences of population levels between experimental time points within the same bacterial species (*p* < 0.05). The values in the parenthesis above the points indicate the results of enrichment tests as positive samples out of total trials (n = 6). The arrow bars (↓) indicate the general feeding interval (4 h) and use-by date of PIF suggested manufacturer (3 weeks).

During storage, cross-contaminated pathogens exhibited noticeable survival on the spoons (Fig. 2) and in PIF (Fig. 3). After typical feeding interval (4 h), bacterial count was 5.3, 4.5, 5.4 log CFU/spoon and 3.6, 2.8, 3.6 log CFU/PIF for *C. sakazakii*, *S*. *enterica*, *S. aureus*, respectively. From 4 to 72 h of storage, surviving populations of *C. sakazakii* and *S*. *enterica* on the spoons decreased significantly (from 5.3 to 4.6, and from 5.2 to 3.7 log CFU/spoon for *C. sakazakii* and *S*. *enterica*, respectively; *p* < 0.05), but *S. aureus* displayed consistent survival during this period (5.2−5.5 log CFU/spoon; *p* > 0.05). Until 2 weeks of storage, although the surviving populations of all tested pathogens had decreased significantly (*p* < 0.05), populations as high as 3.5, 2.4 and 3.8 log CFU/spoon remained viable for *C. sakazakii*, *S*. *enterica* and *S. aureus*, respectively. Populations on the spoons after 3 weeks of storage were 2.6, 1.6, and 3.2 log CFU/spoon for *C. sakazakii*, *S*. *enterica*, and *S. aureus*, respectively.

Regarding bacterial survival in PIF (Fig. 3), populations of *C. sakazakii* and *S. aureus* were clearly resistant to the environmental conditions during storage, since the population up to 72 h oscillated between 3.1–3.5 (*C. sakazakii*) and 3.4–3.6 log CFU/PIF (*S. aureus*). After 2 weeks, bacterial populations of *C. sakazakii* and *S. aureus* gradually decreased over time, reaching 1.9 and 1.5 log CFU/PIF after 3 weeks, respectively. In the case of *S*. *enterica*, bacterial cell counts did not significantly decrease from 4 h (2.8 log CFU/PIF) to 72 h (2.3 log CFU/PIF; *p* > 0.05) but were reduced to 0.3 log CFU/PIF after 2 weeks of storage. After 3 weeks, populations of *S*. *enterica* decreased below the detection limit (<1.7 log CFU/PIF) but could be recovered by enrichment from all trials (n = 6).

### Cross-contamination and survival during handling and storage of PIF following contact with a moderate bacterial load on skin

Following contact with a moderate bacterial load on artificial skin (2.9–3.0 log CFU/hand), contamination on the spoons was evident with a bacterial population of 2.3–2.4 log CFU/spoon (transfer ratio = 80.3–84.0%). However, the pathogen levels in PIF were below the detection limit (<1.7 log CFU/PIF) in all replicates (n = 6). Among the 20 replicate trials for qualitative analysis, 20, 17, and 19 positive results were obtained for *C. sakazakii*, *S*. *enterica*, and *S. aureus*, respectively.

**Figure 4:**
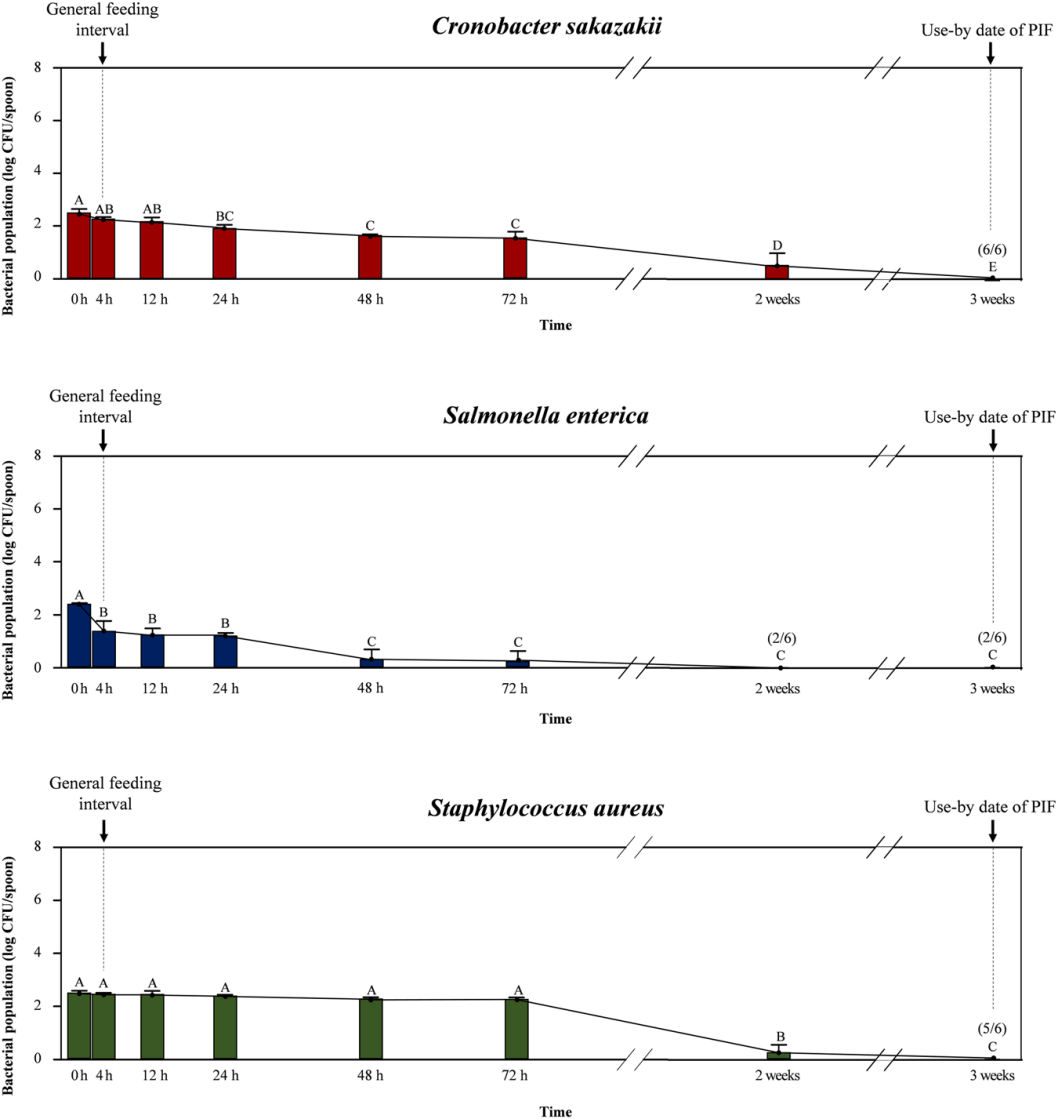
Survival of bacteria (log CFU/spoon) on surface of spoon contaminated from artificial skin with moderate bacterial load (3 log CFU/hand) during storage in touched with PIF. Data showed mean ± standard deviation (quantitative analysis; n = 6). Different capital alphabets (**A**–**E**) denoted on each data indicate significant differences of population levels between experimental time points within the same bacterial species (*p* < 0.05). The values in the parenthesis above the points indicate the results of enrichment tests as positive samples out of total trials (n = 6). The arrow bars (↓) indicate the general feeding interval (4 h) and use-by date of PIF suggested manufacturer (3 weeks).

Survival of cross-contaminated pathogens on the spoons (initial bacterial concentration = 2.4–2.5 log CFU/spoon) is shown in Fig. 4. During the first 72 h of storage, *C. sakazakii* decreased gradually from 1.5 to 2.2 log CFU/spoon, whereas differences in *S. aureus* counts (2.2–2.5 log CFU/spoon) were not significant (*p* > 0.05). While *S*. *enterica* displayed a significant reduction after 4 h (1.4 log CFU/spoon; *p* < 0.05) and reached 0.2 log CFU/spoon at 72 h. Populations of all tested pathogens declined considerably after 2 weeks of storage (1.0 and 0.2 log CFU/spoon for *C. sakazakii* and *S. aureus*, respectively), and only *S*. *enterica* fell below the detection limit (<1.7 log CFU/PIF) but was recovered in two out of six qualitative analysis replicates. The presence of cross-contaminated pathogens on the spoons was detected up to 3 weeks (six, two, and five positive results out of six replicates for *C. sakazakii*, *S*. *enterica*, and *S. aureus*, respectively).

Since the number of vegetative cells in PIF could not be determined by quantitative analysis, the presence of bacteria was examined by recovery from enriched samples (Table 2). *C. sakazakii* and *S. aureus* survived for 3 and 2 weeks, respectively. *C. sakazakii* displayed the highest survival rate, yielding positive results from five positive results after 3 weeks. *S*. *enterica* exhibited the lowest survival rate and remained viable up to 48 h in two replicates. *S. aureus* was recovered from PIF after 2 weeks of storage (four and two positive results after 72 h and 2 weeks of storage, respectively).

**Table 2:** Qualitative microbiological analysis for the bacterial survival in powdered infant formula (PIF) contaminated via spoon from artificial skin with moderate bacterial load during the storage.

Time	No. of positive samples in PIF at moderate bacterial load on hand^x^		
	*Cronobacter sakazakii*	*Salmonella enterica*	*Staphylococcus aureus*
0 h	++++++	++++	+++++
4 h	++++++	++++	+++++
12 h	++++++	+++	+++++
24 h	++++++	+++	+++++
48 h	++++++	++	+++++
72 h	+++++	ND^y^	++++
2 weeks	+++++	ND	++
3 weeks	+++++	ND	ND

^x^Bacterial cells were recovered by the enrichment of samples in six replications (+: growth on corresponding agar).

^y^ND: Not detected.

### Cross-contamination and survival during handling and storage of PIF following contact with a low bacterial load on skin

Since the bacterial population on the spoons and in PIF could not be detected using the direct-plate method (detection limit = 0.7 log CFU/spoon, 1.7 log CFU/PIF), this was assessed as the number of positive results from enrichment tests (20 replicates; Table 1). The initial level of pathogens on artificial skin was 0.9–1.0 log CFU/hand. Following contamination of the spoons by artificial skin, 19, 18, and 19 of positive results were obtained for *C. sakazakii*, *S*. *enterica*, and *S. aureus*, respectively. In the case of PIF, the number of contaminated samples was noticeably low (three and two positive results for *C. sakazakii* and *S. aureus*, respectively), and no cross-contamination of *S*. *enterica* was evident (i.e. no positive results out of 20 replicates).

During subsequent storage of PIF, none of the pathogens were detected after 4 h of storage (i.e. no positive results out of six replicates). However, *C. sakazakii* and *S. aureus* on the spoons were detected up to 2 weeks after enrichment (two and one positive results out of six replicates, respectively), and *S*. *enterica* could be recovered up to 72 h (one positive result out of six replicates; Table 3).

**Table 3:** Qualitative microbiological analysis for the bacterial survival on the surface of spoon contaminated from artificial skin with low bacterial load during storage with powdered infant formula.

Time	No. of positive samples on spoon from low bacterial load on skin^x^		
	*Cronobacter sakazakii*	*Salmonella enterica*	*Staphylococcus aureus*
0 h	++++++	+++++	++++++
4 h	++++++	+++++	+++++
12 h	+++++	+++	+++++
24 h	+++++	+++	++++
48 h	+++	++	++
72 h	++	+	++
2 weeks	++	ND^y^	+
3 weeks	ND	ND	ND

^x^Bacterial cells were recovered by the enrichment of samples in six replications (+: growth on corresponding agar).

^y^ND: Not detected.

## Discussion

There have been survey researches regarding the consumers’ unhygienic practice which can be an immediate cause of cross-contamination during the handling of PIF^9,14,16^. However, although human skin and utensils are generally known to act as reservoirs of pathogens^18,26^, the probability of bacterial transfer from food contact surfaces to PIF has been underestimated. Previous studies focused on the bacterial level present on feeding utensils^27,28^, and to our knowledge, the present study is the first to reveal the occurrence of cross-contamination during PIF handling by caregivers as an extrinsic route (Supplementary Fig. S1).

Research on the cross-contamination between surfaces has revealed that the transfer of bacteria varies due to the difficulty of managing environmental factors^22,29,30^. To minimize differences between laboratory experiments and real-life conditions, we conducted a simulative analysis of experimental conditions that could affect the results (i.e. surface properties of skin, contact time of skin with spoon, pressure of hand for holding spoon) were strictly controlled.

Our results imply the following: (1) bacterial transfer from hands to PIF via utensils occurred readily, and the risk of cross-contamination was strongly dependent on the bacterial load on hands; (2) survival of transferred pathogens was dependent on the type of bacteria; (3) long-term survival of pathogens on spoons and in PIF represents a direct hazard to infants; (4) transmission of pathogens from extrinsic contamination routes should be considered for quantitative microbiological risk assessment (QMRA); and (5) storage of spoons in PIF containers should be avoided to prevent re-contamination of transferred pathogens on food contact surfaces.

In this study, the bacterial transfer was evaluated at three different bacterial loads (6, 3, and 1 log CFU/skin for high, moderate and low load, respectively) to reflect variation in contamination levels on hands^31^. With a high bacterial load on skin, all tested pathogens were transferred to the spoons at >5 log CFU/spoon (5.2–5.5 log CFU/spoon) and could be subsequently transferred to PIF at >3 log CFU/PIF (3.3–3.7 log CFU/PIF). At moderate load, all pathogens on skin were transferred to the spoons at 2.3–2.4 log CFU/spoon, and successive cross-contamination to PIF was also observed (17–20 positive samples out of 20 enrichment tests). However, only *C. sakazakii* and *S. aureus* were transferred to PIF at low bacterial load on skin (three and two positive samples, respectively, out of 20 enrichment tests). Although the transfer ratio of bacteria followed a negative linear trend with initial bacterial load (e.g. transfer ratio from artificial skin to spoons was 24.5–38.1% at a high load, compared with 80.3–84.0% at a moderate load), this negative correlation between transfer ratio and inoculation level is a common phenomenon^30,32^ because the number of bacteria transferred by contact was not lifted proportionally to the increase of inoculation level^33^. Since the impact on infection was determined by exposure dose of pathogens rather than transfer ratio, population level should be a major index for assessing the risk of cross-contamination.

Bacterial species was also identified as major factor determining cross-contamination and survival of pathogens. Since the experimental conditions were strictly controlled in the simulative analysis, the degree of bacterial transfer and survival could be accurately determined for each bacterial species. Previous research showed that surface-to-surface bacterial transfer is affected by intrinsic factors such as the physiological characteristics of bacteria, their degree of attachment, and their clustering capacity^34^. Our results did not reveal significant differences in bacterial transfer (*p* > 0.05; except for population levels on spoons following exposure to a high bacterial load on skin) regardless of bacterial species, even though factors contributing to bacterial transfer including the formation of clumps for *S. aureus*^22^ and the capacity of attachment to inert surfaces for *Salmonella*^35,36^ have been reported. Although simulative research on cross-contamination of *C. sakazakii* has not been conducted previously, outstanding attachment capacity and production of exopolysaccharides are likely to promote bacterial transfer^33,37,38^. Meanwhile, survival on surfaces (both spoons and PIF) was more pronounced for *S. aureus* and *C. sakazakii* than *S. enterica*, highlighting differences in the tolerance of desiccation and limited nutrients. Tolerance to desiccation and long-term survival in low-moisture environments have been reported for *C. sakazakii*^39^*, S. aureus*^40,41^ and *S. enterica*^42^. However, differences in the composition of membrane between Gram-positive bacteria (*S. aureus*) and Gram-negative bacteria (*C. sakazakii, S. enterica*) especially for thicker peptidoglycans of Gram-positive bacteria are expected to support the higher desiccation tolerance^43–45^. Relatively low survivability of *S. enterica* is likely due to the outstanding resistance of *C. sakazakii* against the desiccation stresses among Gram-negative bacteria^46–48^ as reported by previous researches regarding the mechanism of long-term survival under drying conditions (e.g. the production of compatible solutes, the expression of genes involved in osmoprotectant synthesis and transport, etc.)^49,50^. Whereas further investigations of other factors determining survival pathogens following cross-contamination from surface-to-surface transfer are also needed to predict the actual risks for each pathogen.

Analysis of bacterial survival revealed that unhygienic practices by the caregivers not only caused cross-contamination, but also resulted in the long-term survival of pathogens in PIF (up to 3 weeks, use-by date suggested by manufacturers). Cross-contamination of pathogens in PIF highlights the risk of infection to infants continually exposed via feeding. Previous research on the survival of pathogens in PIF involved direct inoculation into the PIF product^51,52^. By contrast, in the present study, survival of bacteria was analyzed in accordance with the general feeding frequency and consumption periods of PIF to identify the actual risk of infection. The results showed that following exposure to a high bacterial load on skin, infants can be directly exposed to pathogens at levels causing foodborne illnesses (i.e. above the minimal infectious dose) through feeding of cross-contaminated PIF^46,53^. Although transfer to PIF was below the detection limit at moderate and low bacterial loads on skin, reconstitution of PIF and handling of hydrated formula milk could allow the multiplication of pathogens, resulting in foodborne illnesses^54^. Parra-Flores, *et al*.^55^ also highlighted the possibility of illness caused by < 1 CFU/g of bacterial cells using a *C. sakazakii* growth model in reconstituted PIF (i.e. hydrated formula milk). Pathogenic *S. aureus* has been reported a causative factor in fatal diseases of infants through the contaminated breast milk, and this study suggests PIF as novel route of contamination^56–58^. Further research on the prediction of risk and countermeasures for pathogen control (e.g. personnel hygiene and thermal inactivation during reconstitution of PIF) should be considered^59^.

The high probability of cross-contamination from extrinsic sources revealed in this study highlights the fact that QMRA models should incorporate cross-contamination scenarios and simulation models as the key route of pathogens in PIF. QMRA estimation of risk based on controlled exposure to pathogens comprises hazard identification, hazard characterization, exposure assessment, and risk characterization^60,61^. When constructing a QMRA framework module, comprehensive consideration on the possible routes of pathogen transmission is important for accurate risk estimation^33,62^. To incorporate cross-contamination in QMRA, transfer characteristics of pathogens have been investigated and/or modeled for various scenarios, including transfer from contaminated slicing machines to cooked meat products^63^, from chicken to cutting boards in kitchens^64^, and from lettuce to knives in kitchens^65^. However, currently available QMRA models for the safe management of PIF underestimate the possibility of cross-contamination from consumers (i.e. only contamination in the manufacturing environment is considered as a pathogen source) due to a lack of background information^66^. Further research on QMRA, especially on the development of framework modules incorporating cross-contamination scenarios and simulation models including bacterial transfer, are needed to develop mitigation strategies against foodborne illnesses^33,34^.

Cross-contamination and bacterial survival involving spoons highlights the importance of hygienic maintenance of feeding utensils. Cross-contamination of pathogens on the spoons was still recoverable after 3 weeks of storage. At low bacterial load, pathogens contaminating PIF did not survive beyond 4 h whereas bacterial survival on the surfaces of spoon was observed after 72 h. This demonstrates the potential for re-contamination and the accumulation of pathogens in PIF following repetitive use of utensils. Therefore, feeding utensils should not be kept in the PIF container to prevent cross-contamination via contact. Instead, spoons should be stored separately from PIF and sterilized thoroughly before use.

The implications of the present study should be perceived and acted on by infant caregivers. Regarding food preparation at home, caregiver’s hands can be contaminated with bacteria present in raw materials (e.g. meat, eggs and fresh produce) and may act as vectors of foodborne pathogens, resulting in cross-contamination^67,68^. Thus current practices of caregivers should be re-evaluated with the perspective to the risk of cross-contamination and the intervention strategies for the prevention of foodborne infectious diseases through hygienic practices are needed^69^. In hospitals, major outbreaks associated with PIF have occurred in neonatal intensive care units^70,71^ and cross-contamination was recognized as a primary cause^72,73^. However, research on caregivers as a source of the PIF contamination is scarce. Postnatal care centers and day-care centers are also prone to microbiological infection due to the preparation of large batches of PIF in advance^12^. Therefore, proper precautions and countermeasures for caregivers should be in place to improve their awareness of cross-contamination and prevent foodborne illnesses^74^.

## Methods

### Bacterial strains and preparation of cell suspensions

*C. sakazakii* (ATCC 12868, 29004, 29544), *S*. *enterica* (Typhimurium ATCC 19585, 14028, Enteritidis ATCC 13076) and *S. aureus* (ATCC 23235, 25923, NRS 111) were obtained from the American Type Culture Collection (Manassas, VA, USA). Each strain was stored at -20°C in Tryptic Soy Broth (TSB; Difco, Sparks, MD, USA) containing 20% glycerol (w/v). A sterile loop was used to inoculate each strain into 3 ml TSB and incubated at 37°C for 24 h. Then cultures were combined in 50 ml conical tubes (Becton Dickinson, Franklin Lakes, NJ, USA), centrifuged at 1,800 × g for 15 min using a CentraCL2 instrument (International Equipment Company, Needham Heights, MA, USA), and the supernatant was discarded. After washing the pellet twice with 0.85% saline, cells were resuspended with the same amount of 0.85% saline, and mixtures were diluted to obtain the required bacterial cell density.

### Preparation of artificial skin

Artificial skin (VITRO-SKIN N-19; IMS Inc., Portland, OR, USA) designed to mimic the surface properties of human skin such as topography, pH, critical surface tension and ionic strength, was used as alternative to bare hands^29,75^. Pieces of artificial skin were cut into 4 cm × 1.5 cm fragments to fit the surface area to the part of the spoon handle in contact with hand, and exposed to 260 nm UV radiation for 1 min at ambient temperature (*ca*. 23°C) under a laminar flow hood for sterilization. To achieve humidity similar with human skin, fragments of artificial skin were hydrated in a chamber containing 350 ml of 15% glycerin at 23°C (±1°C) for 16–24 h (relative humidity inside in chamber was 80–90%).

Prior to experiments, artificial skin was confirmed to be free from the tested bacteria by enrichment tests as recommended by US Food and Drug Administration (FDA) and International Organization for Standardization (ISO) with some modifications^76–80^. The surface of artificial skin was swabbed with two cotton sticks (one swab moistened with saline and one dry swab), and the heads of the cotton sticks were placed in a 15 ml conical tube (Falcon Becton Dickinson, Franklin Lakes, NJ, USA) containing 10 ml broth medium specific for each bacterium and incubated under appropriate conditions for bacterial growth [*C. sakazakii*: buffered peptone water (BPW) at 37°C for 24 h, then 0.1 ml culture + 10 ml Cronobacter Screening Broth supplemented with 10 μg/ml vancomycin at 37°C for 24 h; *S. enterica*: BPW at 37°C for 24 h, then 0.1 ml culture + 10 ml Rappaport-Vassiliadis medium at 42°C for 24 h; *S. aureus*: TSB supplemented with 10% NaCl and 1% sodium pyruvate at 35°C for 24 h]. Enriched samples were then separately streaked and incubated on selective agar, and typical colonies were confirmed as follows: blue-green colonies on Druggan-Forsythe-Iversen agar (DFI; Oxoid) after incubation at 37°C for 24 h for *C. sakazakii*; black colonies on xylose-lysine-desoxycholate agar (XLD; Difco) after incubation at 37°C for 24 h for *S. enterica*; and yellow colonies with precipitation zones on mannitol salt agar (MSA; Difco) supplemented with 5% egg yolk after incubation at 35°C for 48 h for *S. aureus*.

### Preparation of spoon and PIF samples

Commercial PIF product was purchased from a local retail store (Seoul, Korea), and spoons (polypropylene; total length = 13.5 cm, length of handle = 8.7 cm, width of handle = 1.5 cm) were enclosed in canisters of PIF. Spoons were washed with detergent and water, then autoclaved at 121°C for 15 min. For each experiment, 5 g of PIF was prepared in a sterile aluminum container (14 cm × 2.5 cm × 2 cm). Before commencement of experiments, PIF used in this study was determined to be free of *C. sakazakii*, *S*. *enterica*, and *S. aureus* through the enrichment test^81,82^ conducted by following qualitative microbiological analysis methods as described above. PIF samples were incubated in enrichment broth (*C. sakazakii*: BPW, Cronobacter Screening Broth supplemented with 10 μg/ml vancomycin; *S. enterica*: BPW, Rappaport-Vassiliadis medium; *S. aureus*: TSB supplemented with 10% NaCl and 1% sodium pyruvate), and then sample homogenates were streaked on selective agar (DFI, XLD, and MSA supplemented with 5% egg yolk for *C. sakazakii*, *S. enterica*, and *S. aureus*, respectively). After the incubation of selective agar plates, we evaluated the absence of target pathogens in PIF samples (i.e. no presumptive colony on selective agar plates).

### Designation of the scenario for the cross-contamination

As shown in Fig. 1, we assumed the scenario as follows: various bacterial loads on caregiver’s hands are transferred to spoons when grasped during scooping of PIF. Cross-contamination then occurs when contaminated spoons contact with PIF in the canister. Pathogen survival on spoons and in PIF can cause infection in infants fed with contaminated PIF.

Simulative analysis was performed to reflect the real-situation and to control the determinant factors of the cross-contamination. To replicate holding the spoon with hand, spoon handle was wrapped in fragments of artificial skin on a balance under constant pressure (0.2 kg/cm^2^) to reflect the typical value applied between the abiotic surface and the hand^83,84^. The duration of holding the spoon was set at 20 s, based on the average period for PIF preparation validated by 100 volunteers. An aluminum container containing PIF was used to simulate storage of spoons. The sampling time for analysis of bacterial survival was determined by considering the general feeding interval (i.e. 6–8 feeds per day for the first few weeks, 4 h), and the typical consumption period of a single PIF product (i.e. 3 weeks, use-by date suggested by the manufacturer)^85^.

### Experimental protocol for the simulative analysis

A 20 μl bacterial suspension was uniformly applied in five droplets onto two fragments of artificial skin and spread for 1 min at three different contamination levels (total bacterial populations on the two fragments were considered representative of bacterial contamination on a single hand); high, moderate, and low bacterial load (*ca*. 6, 3, and 1 log CFU/hand, respectively)^31^. Inoculated fragments were placed on a petri dish in a laminar flow biosafety hood for 5 min to dry. Then one fragment was placed below a spoon handle and the surface of the handle was covered with the other fragment with pressure (0.2 kg/cm^2^ for 20 s). The spoon was then transferred to an aluminum container and the handle of the spoon was covered with 5 g of PIF. The initial bacterial load, and the cell density of transferred bacteria on the spoon and on PIF were examined immediately after inoculation, following contact of spoon with skin fragment, and covering of the spoon with PIF. Quantitative microbiological analysis of the three different bacterial loads was replicated six times. Enrichment tests (20 replications) for qualitative microbiological analysis were conducted to verify transmission if bacteria transferred to the recipient surface could not be detected by quantitative analysis (detection limit = 0.7 log CFU/spoon, 1.7 log CFU/PIF).

To simulate bacterial survival during subsequent storage, containers containing spoons and PIF were placed in a larger polypropylene container with a lid (145 mm × 95 mm × 49 mm; Lock&Lock, Seoul, Korea) and stored at 23°C (±1°C) in the incubator. Surviving bacteria on spoons and PIF were counted immediately (0 h) and after storage for 4, 24, 48 and 72 h, and 2 and 3 weeks. Both quantitative and qualitative analyses (six replications) for one spoon and 5 g PIF were analyzed at each time point.

### Quantitative microbiological analysis

The surface swab method (swab for 30 s with two cotton sticks, one moistened with saline and one dry) was employed to measure bacterial cell density on the surface of artificial skin fragments and spoons^29^. Heads of cotton sticks were aseptically cut and placed in 5 ml 0.85% saline for 15 min, then vortexed for 30 s. PIF (5 g) was aseptically placed in a stomacher bag (Seward, UK) with 45 ml 0.85% saline and homogenized using a stomacher (Circulator 400 standard bas; Seward, Worthing, UK) at 230 rpm for 2 min. A 1 ml sample was serially diluted 10-fold in 9 ml 0.85% saline, and 0.1 ml aliquots were spread on each selective agar. To lower the detection limit, 0.2 ml of each suspension was spread on five plates. After incubation at 37°C for 24 h, typical colonies were enumerated. The results were expressed as log CFU/hand, log CFU/spoon, and log CFU/PIF for artificial skin, spoons, and PIF, respectively (detection limit = 0.7 log CFU/spoon and 1.7 log CFU/PIF, respectively). Transfer ratio from artificial skin to spoons, or from spoons to PIF, was defined as the bacterial cell population level recovered from the recipient surface (i.e. spoons or PIF) relative to that recovered from the donor (i.e. artificial skin or spoons)^86^, and was calculated as follows:

$${\rm{Transfer}}\,{\rm{ratio}}\,( \% )=\frac{{\rm{CFU}}\,{\rm{recipient}}}{{\rm{CFU}}\,{\rm{donor}}}\times 100$$

### Qualitative microbiological analysis

Enrichment tests were conducted as described above. In brief, cotton sticks swabbed against spoons were cut and placed in enrichment medium. PIF (5 g) was transferred to a stomacher bag (Seward) containing 45 ml enrichment medium and homogenized with a stomacher (Seward) for 2 min at 230 rpm. Samples were incubated under conditions appropriate for each pathogen, then streaked onto selective agar plates.

### Statistical analysis

The population level of each pathogen from duplicate average plate counts (six replicates) was converted into a logarithmic value. All experimental data (population level, transfer ratio) were expressed as means ± standard deviation. Analyses for difference between data were performed using the general linear model (GLM) procedure with SAS software version 9.4 (SAS Institute Inc., Cary, NC, USA). The significance of differences was assessed using Tukey’s multiple comparison tests, and the significance level was set at a *p*-value of <0.05. In Figs 2–4, Significant differences of population levels between experimental time points within the same bacterial species were denoted by different capital alphabets on data.

## Supplementary information

Supplementary Figure 1

## Data Availability

All data generated or analyzed during this study are included in this published article (and its Supplementary Information files).
