# Supplementary material for: Underestimated Risks of Infantile Infectious Disease from the Caregiver’s Typical Handling Practices of Infant Formula
Source: Sci Rep. 2019 Jul 5;9:9799. doi: 10.1038/s41598-019-46181-0 (PMC6611816; doi:10.1038/s41598-019-46181-0)
Supplement: Supplementary file 1 — Supplementary Figure 1 [file 41598_2019_46181_MOESM1_ESM.docx]

**Supplementary Information**

**Manuscript title**

Underestimated Risks of Infantile Infectious Disease from the Caregiver’s Typical Handling Practices of Infant Formula

**Author list**

Tae Jin Cho, Ji Yeon Hwang, Hye Won Kim, Yong Ki Kim, Jeong Il Kwon, Young Jun Kim, Kwang Won Lee, Sun Ae Kim, and Min Suk Rhee

**Supplementary Fig. 1.** Summary of major findings and implications of the present research.

**Supplementary Fig. 1**

**
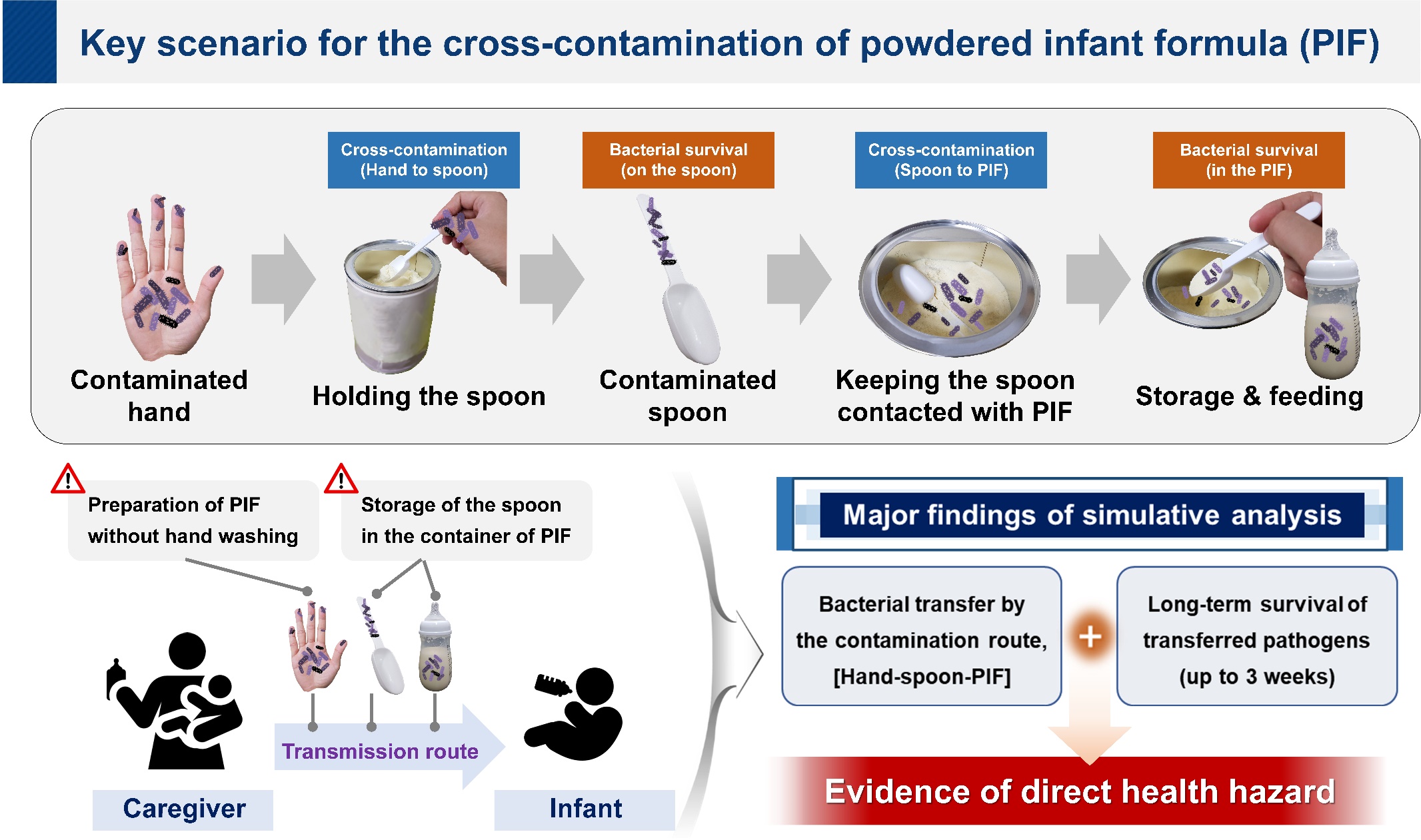
**
